# Vessel Scheduling Optimization Model Based on Variable Speed in a Seaport with One-Way Navigation Channel

**DOI:** 10.3390/s21165478

**Published:** 2021-08-14

**Authors:** Dongdong Liu, Guoyou Shi, Katsutoshi Hirayama

**Affiliations:** 1 Department of Navigation College, Dalian Maritime University, Dalian 116026, China; liudongdongdmu@dlmu.edu.cn; 2 Key Laboratory of Navigation Safety Guarantee of Liaoning Province, Dalian 116026, China; 3 Department of Global Transportation Sciences, Kobe University, Kobe 658-0022, Japan; hirayama@maritime.kobe-u.ac.jp

**Keywords:** vessel scheduling problem, maritime shipping, optimal scheduling, variable speed, time windows

## Abstract

To improve the efficiency of in-wharf vessels and out-wharf vessels in seaports, taking into account the characteristics of vessel speeds that are not fixed, a vessel scheduling method with whole voyage constraints is proposed. Based on multi-time constraints, the concept of a minimum safety time interval (MSTI) is clarified to make the mathematical formula more compact and easier to understand. Combining the time window concept, a calculation method for the navigable time window constrained by tidal height and drafts for vessels is proposed. In addition, the nonlinear global constraint problem is converted into a linear problem discretely. With the minimum average waiting time as the goal, the genetic algorithm (GA) is designed to optimize the reformulated vessel scheduling problem (VSP). The scheduling methods under different priorities, such as the first-in-first-out principle, the largest-draft-vessel-first-service principle, and the random service principle are compared and analyzed experimentally with the simulation data. The results indicate that the reformulated and simplified VSP model has a smaller relative error compared with the general priority scheduling rules and is versatile, can effectively improve the efficiency of vessel optimization scheduling, and can ensure traffic safety.

## 1. Introduction

**Figure 1:**
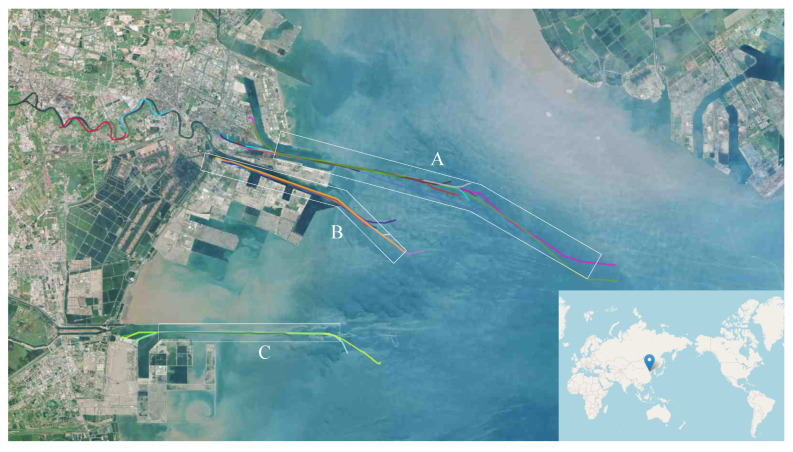
Research port area and the trajectory of in-wharf vessels and out-wharf vessels.

The port management department manages the entry and exit operations of most ships through a service with a fixed timetable. With the development of large-scale ships, the capacity of ships in port waters has gradually become restricted. For example, in the port of Tianjin shown in Figure 1, formerly known as the port of Tanggu, all ship movements and major operations must be consistent with the directive requirement of the Tianjin Port Group’s Operations Department. When sailing on the main fairway that is area A in Figure 1, the under-keel clearance (UKC) shall not be less than 1.7 m. When one-way navigation is applied, a vessel shall keep a safe distance of more than six times her own vessel’s length with other vessels. Therefore, the control of the vessel traffic requires further consideration of many factors involving the depth of the port waters, the height of the tide, the ship’s draft, the time or speed of the ship entering and leaving the port, the estimated time of arrival (ETA), etc.

To overcome the problem of low manual scheduling efficiency, some works had been presented from the view of the reduction of occupied time in berth, anchorage, or the channel. Many studies focus on increasing navigation efficiency in inland ports, seaports, and waterways through applying simulation techniques. Some of these works adopt the idea of collision-free routing to control the traffic at a canal. Günther, E. et al. [1,2] designed a successive shortest path algorithm respecting blocked time windows to solve the optimization problem of ship traffic control at the Kiel Canal by constructing conflict-free dynamic routes for the ships one after another. Lübbecke, E. et al. [3] further integrate two algorithms that address collision avoidance to solve the combinatorial optimization problem of ship traffic control at the Kiel Canal: one is train scheduling on a single-track railway network and the other is collision-free routing for automated guided vehicles. Moreover, a fast heuristic was developed which can make the average time consumed be less than two minutes to plan all ships for one day. Meisel, F. [4] and Skjæveland, G. [5] extended the MIP model of Lübbecke, E. et al. [3] by deciding speeds of vessels rather than assuming constant given speeds. Andersen, T. et al. [6] further extended the previous works on traffic control of the Kiel Canal considering the uncertainty characteristic of the time of arrival at the entrance to the canal. Moreover, some other works focus on heuristic algorithms and mathematical models to optimize the navigation plan. Muñuzuri, J. [7] utilized an exhaustive search algorithm for the optimal scheduling at inland waterways subject to tidal variations in depth. Considering the effect of the periodic tide, Kelareva, E. [8] proposed CP and MIP methods for ship scheduling. Zhang, B. [9,10] established two scheduling models for the vessel through the one-way channel and compound channel, respectively, with a genetic algorithm (GA). Most research on vessel scheduling problems (VSP) concerns how to obtain a baseline schedule in a static and deterministic environment with complete information [11,12,13].

Studies on the vessel transportation scheduling solution for entering and leaving ports have largely focused on a single aspect of channel usage efficiency or berth operation. However, few have considered coordination between channel and berth resource demands. With regard to this problem, Guo Zijian et al. [14] proposed an integrated scheduling model which can simultaneously optimize the vessel sequence, lay-by berth allocation, and de-ballasting plan. The integrated optimization model of the berth allocation problem (BAP) and VSP in a seaport with a one-way navigation channel were constructed by Zhang, X. et al. [15] and Liu, B. et al. [16]. Zhang, X. et al. [17] also reduced the total scheduling time by more than 40% for a one-way port-channel unitizing multi-objective genetic algorithm (MOGA). Based on an analysis of the navigation mode and the traffic conflicts in a compound waterway, the optimization model was expanded to solve the VSP in a compound waterway of a large harbor by Zhang, X. [18]. Lalla-Ruiz et al. [19] introduced the waterway ship scheduling problem (WSSP) where the goal is to minimize the ship’s waiting time on the Yangtze Estuary (Shanghai) with greedy heuristics (GH) and simulated annealing (SA). Hill, A. and Lalla-Ruiz et al. [20] reconstructed the ship scheduling model under multi-mode resource constraints which considered the management of inbound and outbound ships. Considering the effect of traffic conflicts in the Huanghua coal port where multi-harbor basins share a restricted channel, Li, J. et al. [21] developed a heuristic algorithm to tackle the mode by integrating the non-dominated sorting genetic algorithm II (NSGA-II) and Tabu Search (TS). Dulebenets, M. A. [22,23] provided a comprehensive multi-objective optimization model to tackle the VSP in liner shipping and summarized critical literature and future research directions. Kang, L. [24] formulated the uncertain ship arrival and tugging times for container ports as a finite set of discrete scenarios and proposed a mixed-integer linear programming model. Ulusçu, Ö. S. [25,26] modeled the waiting time approximation in queueing systems with multi-types of class-dependent interruptions. Except for this, all the other studies are the deterministic models for tugboat scheduling optimization [27,28,29,30,31].

**Figure 2:**
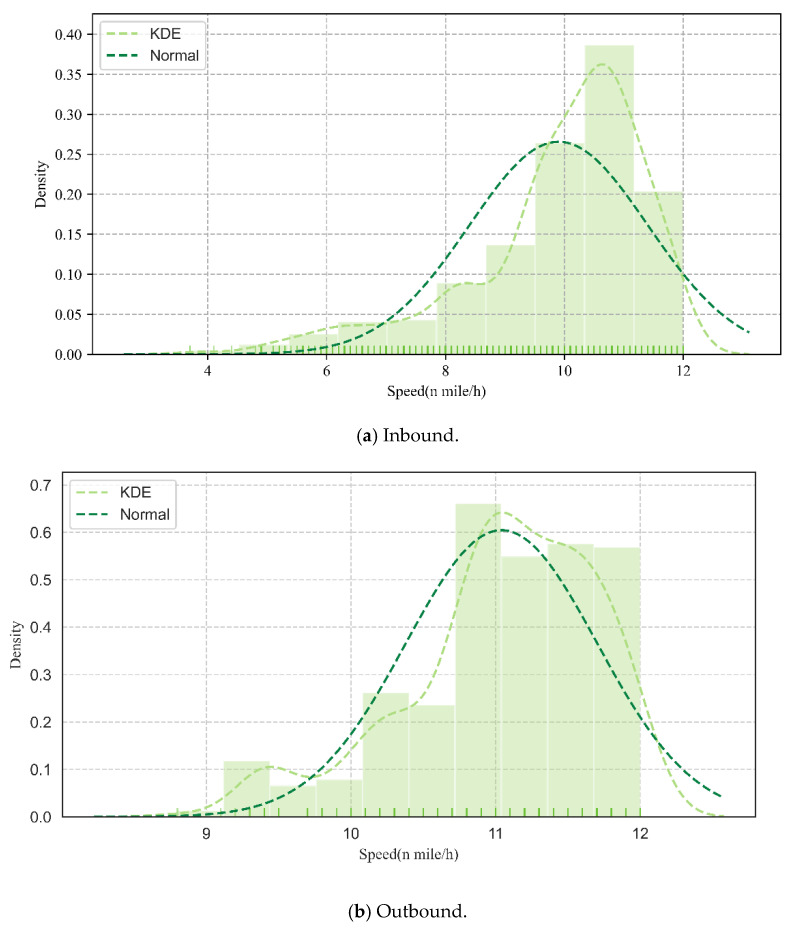
Speed distribution of in-wharf vessels and out-wharf vessels in March 2015. Where the lighter dashed line represents the kernel density distribution curve of speed, the darker dotted line indicates the normal distribution curve of speed, the short solid line indicates the statistical speed data, and the histogram indicates the density distribution of speed intervals.

In summary, the current vessel sequence arrangement operations ignore variables in realistic situations such as the sailing speed through the channel, equipment failures, and other unforeseen events. These previous studies listed in Table 1 did not consider that the sailing speed is not fixed, shown in Figure 2, during the implementation of the optimal plan; therefore, the optimal result based on the constant speed cannot clearly represent the situation of practical implementation. Thus, the major objective of this study is to tackle the vessel scheduling optimization problem based on variable speed in a one-way navigation channel. However, different from the above-mentioned deterministic optimization work [32], there are many theories about uncertainty optimization and they have been well-applied in other fields, such as fuzzy programming, stochastic optimization, and robust optimization [33]. For the VSP under uncertainty, all of the above uncertain theories can be utilized to model the problem from different perspectives. In this study, the vessel scheduling optimization problem based on variable speed in a one-way navigation channel was tackled by extending the local safety distance constraint problem at the entrance and exit of the channel to the global safety distance constraint problem for the entire channel.

The following studies consist of five sections. Section 2 states the vessel scheduling problem and the whole operation process as well as different encounter situations in a one-way waterway. Section 3 introduces the assumptions and limitations for the proposed VSP model and develops the mathematical model of vessel scheduling under indeterminate speed. Besides, a concept of Minimum safety time interval (MSTI) is also proposed in Section 3 to reduce time constraints by integrating multi-time constraints. A heuristic algorithm GA to optimize the scheduling solution is designed in Section 4. In Section 5, numerical experimental analysis is carried out to compare the efficiency between different methods for optimal scheduling and verify the effectiveness of the proposed VSP model. Section 6 concludes the attribution of this study and the future work.

## 2. Problem Settings

**Figure 3:**
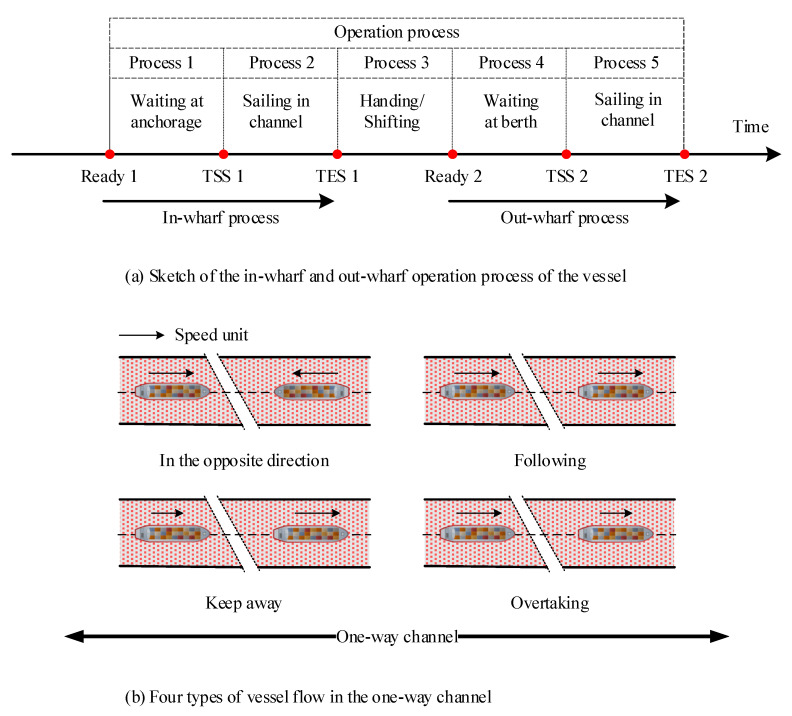
Schematic diagram of the ship operation process and traffic flow.

Vessel scheduling optimization aims to arrange the sequence of in-wharf vessels and out-wharfs vessels that change in real-time while reducing the waiting time of vessels and the occupancy rate of resources such as waterways, anchorages, and berths as much as possible [34]. In the actual production process of the port, vessel scheduling includes three major resources. The most concerned is the channel and berth resource optimization problem. In a one-way waterway, there are four types of encounter situations including following, keep away, overtaking, and head-to-head shown in Figure 3b. Due to the fact that the waterway cannot be shared by more than one vessel, another vessel needs to wait out of the waterway until the waterway is clear because there exists a conflict, which is referred to as the head-to-head situation shown as in Figure 3b. The encounter type of keeping away means that the speed of the rear vessel is slower than the front vessel in the same direction, and the other encounter situations are also differentiated based on the direction and magnitude of the speed. Generally, the speed of in-wharf vessels and out-wharf vessels does not remain constant; therefore, the related research just considering the fixed speed [9,10,14,15,16,17,18] will be difficult to apply to actual production. In reality, constrained by different operation tasks, vessels usually enter or exit the channel from different waters during both the in-wharf process and out-wharf process [33]. Therefore, the position of entering or exiting the channel is not fixed, which would be different with different operation tasks. For convenience, as shown in Figure 3b, the one-way channel studied in this research was assumed to have the same position for entering the channel from anchorage, open sea, or berth or exiting the channel in the above three waters.

In the actual production process of the port, the whole operation process can be divided into 5 parts as shown in Figure 3a, which includes multi-time constraints such as the estimated time of arrival (ETA), time of start scheduling (TSS), time of end scheduling (TES), delaying time (DT), and navigable time windows (NTW) with the tidal constraint. The vessel can enter the channel until the allotted time (TSS 1 or TSS 2), and before this, the vessel needs to wait at the anchorage, open sea, or berth from ETA to TSS (Process 1 or Process 4). Therefore, what we need to determine is the TSS of vessels according to the related time constraint considering the requirement of safe distance for adjacent vessels, and then determine the assigned order of vessels based on TSS.

## 3. Vessel Scheduling Optimization Model Formulation

### 3.1. Assumptions and Limitations

Considering the actual situation of port production, a series of assumptions are defined as follows:

- There are sufficient berths and anchorage resources, and only the issue of ship entry and exit sequence is considered.
- All the relevant time parameters of ships entering and leaving the port are known in advance, and the trajectory in the channel is also known [16].
- The channel has only one entry position and one exit position where no overtaking is allowed.
- We assume that the safe navigation distance between ships in the same direction is six times the length of the ship behind, and the safe threshold is 12 min in the opposite direction [15,17,18].

### 3.2. Vessel Scheduling Optimization Model

One-way channel ‘A’ in Figure 1 is an example which is used to illustrate the optimization process of vessel scheduling. We assume that there are two in-wharf vessels and three out-wharf vessels. The in-wharf vessels are labeled 1 and 2, while the out-wharf vessels are labeled 3, 4, and 5, respectively. Table 2 is a sample of a vessel plan which includes the identifiers (e.g., ship name, mmsi, imo, call sign), module parameters (e.g., length, width, draft), information about entry or exit from the seaport (e.g., estimated time of arrival, start time, end time, state, etc.), and some other relevant information. Actually, in practice, before the plan is scheduled, we only know some key information from Table 2 as listed in Table 3, which involves the environment information as shown in Table 2, the movement types, and some other information about entry or exit from the seaport.

Table 4 is a brief analysis of the vessel schedules of the two in-wharf vessels and three out-wharf vessels with 120 kinds of schedule solutions for VSP optimization. Furthermore, if there are in-wharf vessels and out-wharf vessels, there would be possible timing sequence combinations based on the theory of arrangement and combination. Therefore, the larger the number of vessels, the longer the number of timing sequences. The enumeration method would not be applicable to solve the problem. The following section specifically proposes a MILP model to optimize the schedule of vessels passing through the one-way waterway under variable speed.

#### 3.2.1. Scheduling Optimization Model for VSP

One general VSP model contains many time parameters; the sets, parameters, and decision variables utilized in the following section are defined in Table 5.

A general VSP model contains many time parameters; for example, the relationship between the time at the end of the channel and the time at the entrance of the channel can be stated as Equation (1).

(1) $$TES_{m,i}=TSS_{m,i}+\Delta_{m,i},\;\forall m\in M,i\in I_{m}.$$

After determining the relevant time parameters, the objective function for vessel waiting is obtained, which is defined as the actual time of start scheduling to enter the conflict areas as shown in Figure 3 in a one-way waterway, minus the estimated time of arrival. Therefore, the total waiting time for all vessels to pass through a one-way waterway is shown as Equation (2).

(2) $$[\mathrm{OBJ}]\;D=\sum_{m\in M}\sum_{i\in I_{m}}\left(TSS_{m,i}-ETA_{m,i}\right),\forall m\in M,i\in I_{m}.$$

Furthermore, the objective function can be defined as Equation (3).

(3) $$[\mathrm{OBJ}]\;\;\min\;\Gamma =\sum_{m\in M}\sum_{i\in I_{m}}\left(TSS_{m,i}-ETA_{m,i}\right)/\sum_{m\in M}I_{m}$$

Equation (4) states the relationship between the estimated time of arrival and the scheduled time.

(4) $$TSS_{m,i}-ETA_{m,i}\geq 0,\forall m\in M,i\in I_{m}$$

The depth safety can be constrained by the time window of the tide shown in Equations (5) and (6), where Equation (5) guarantees that the time at the entrance of any channel is greater than the lower bound of the time window. Formula (6) ensures the time at the end of the final channel in the channel chain is less than the upper bound of the time window.

(5) $$TSS_{m,i}-TW_{m,i}^{l}\geq 0,\forall m\in M,i\in I_{m}.$$

(6) $$TW_{m,i}^{r}-TES_{m,i}\geq 0,\forall m\in M,i\in I_{m}.$$

The safety distance between adjacent vessels in the same direction can be stated by Equations (7) and (8), which state the relationship of the entrance time and the exit time of two adjacent vessels in the schedule in the same direction.

(7) $$TSS_{m,i}-TSS_{m,i-1}\geq t_{1},\forall m\in M,i\in I_{m}.$$

(8) $$TES_{m,i}-TES_{m,i-1}\geq t_{1},\forall m\in M,i\in I_{m}.$$

To transform the formula into a normal style that can be optimized by a commercial solver, Equations (7) and (8) can be transformed as Formulas (9)–(11). Constraint (11) ensures Equations (9) and (10) can have the same sequential relationship between two vessels sailing at the entrance and end of the channel.

(9) $$\left|TSS_{m,i}-TSS_{m,j}\right|\geq t_{1},\forall m\in M,i,j\in I_{m}.$$

(10) $$\left|TES_{m,i}-TES_{m,j}\right|\geq t_{1},\forall m\in M,i,j\in I_{m}.$$

(11) $$\left(TES_{m,i}-TES_{m,j}\right)\times \left(TSS_{m,i}-TSS_{m,j}\right)>=0,\forall m\in M,i,j\in I_{m}.$$

Because these Formulas (9)–(11) are non-linear, we must further transform them according to the linear constraint by using 0–1 variables as Formulas (12) and (13).

(12) $$\left\{\begin{array}{cc} TSS_{m,i}-TSS_{m,j}\geq t_{1}, & if\;B_{m,i,j}=1\\ TSS_{m,j}-TSS_{m,i}\geq t_{1}, & if\;B_{m,i,j}=0 \end{array}\right.,\forall m\in M,i,j\in I_{m},i\neq j.$$

(13) $$\left\{\begin{array}{cc} TES_{m,i}-TES_{m,j}\geq t_{1}, & if\;B_{m,i,j}=1\\ TES_{m,j}-TES_{m,i}\geq t_{1}, & if\;B_{m,i,j}=0 \end{array}\right.,\forall m\in M,i,j\in I_{m},i\neq j.$$

Equations (12) and (13) can be further expressed as Formulas (14)–(17) by introducing a sufficiently large positive constant M.

(14) $$B_{m,i,j}\times M+TSS_{m,j}-TSS_{m,i}\geq t_{1},\forall m\in M,i,j\in I_{m},i\neq j.$$

(15) $$\left(1-B_{m,i,j}\right)\times M+TSS_{m,i}-TSS_{m,j}\geq t_{1},\forall m\in M,i,j\in I_{m},i\neq j.$$

(16) $$B_{m,i,j}\times M+TES_{m,j}-TES_{m,i}\geq t_{1},\forall m\in M,i,j\in I_{m},i\neq j.$$

(17) $$\left(1-B_{m,i,j}\right)\times M+TES_{m,i}-TES_{m,j}\geq t_{1},\forall m\in M,i,j\in I_{m},i\neq j.$$

After multi-step conversion, Formulas (9) and (10) are linearized into formulas (14)–(17). Constraints (14) and (15) guarantee that the time interval of any two vessels scheduled at the entrance of a channel is greater than the safe time interval $t_{1}$ in the same direction. Constraints (16) and (17) ensure the time interval of any two vessels scheduled at the end of a channel is greater than the safe time interval $t_{1}$ in the same direction.

The safety distance between adjacent vessels in the opposite direction can be stated by constraint (18), which is to ensure the safety between any vessels in the opposite direction. Adopting the same conversion theory as Formulas (12) and (13), the nonlinear constraints can be transformed into linear constraints as formulas (19, 20) by introducing a 0–1 variable $I_{k,l}$. $I_{k,l}=0$, which means the vessel $k,k\in I_{1}$ precedes $l,l\in I_{2}$, 1 otherwise.

(18) $$\left\{\begin{array}{l} TSS_{1,k}-TES_{2,l}\geq t_{2},if\;I_{k,l}=1\\ TSS_{2,l}-TES_{1,k}\geq t_{2},if\;I_{k,l}=0 \end{array}\right.,\forall k\in I_{1},\forall l\in I_{2}.$$

(19) $$I_{k,l}\times M+TSS_{2,l}-TES_{1,k}\geq t_{2},\forall k\in I_{1},\forall l\in I_{2}.$$

(20) $$\left(1-I_{k,l}\right)\times M+TSS_{1,k}-TES_{2,l}\geq t_{2},\forall k\in I_{1},\forall l\in I_{2}.$$

Since the time decision variable has a serious impact on optimization efficiency, an upper limit is set for the time at the end of a channel shown as Constraint (21), where θ is greater than 1.

(21) $$ETA_{m,i}+\theta \Delta_{m,i}\geq TES_{m,i}\geq ETA_{m,i}+\Delta_{m,i}\;,\forall m\in M,i\in I_{m}.$$

Above all, the general scheduling of the in-wharf and out-wharf vessels consists of a mixed-integer linear programming model shown as in Formula (22).

(22) $$\begin{array}{l} \min\Gamma =\sum_{m\in M}\sum_{i\in I_{m}}\left(TSS_{m,i}-ETA_{m,i}\right)/\sum_{m\in M}I_{m}\\ s.t.TES_{m,i}=TSS_{m,i}+\Delta_{m,i},\;\forall m\in M,i\in I_{m}.\\ TSS_{m,i}-ETA_{m,i}\geq 0,\forall m\in M,i\in I_{m}.\\ TSS_{m,i}-TW_{m,i}^{l}\geq 0,\forall m\in M,i\in I_{m}.\\ TW_{m,i}^{r}-TES_{m,i}\geq 0,\forall m\in M,i\in I_{m}.\\ B_{m,i,j}\times M+TSS_{m,j}-TSS_{m,i}\geq t_{1},\forall m\in M,i,j\in I_{m},i\neq j.\\ \left(1-B_{m,i,j}\right)\times M+TSS_{m,i}-TSS_{m,j}\geq t_{1},\forall m\in M,i,j\in I_{m},i\neq j.\\ B_{m,i,j}\times M+TES_{m,j}-TES_{m,i}\geq t_{1},\forall m\in M,i,j\in I_{m},i\neq j.\\ \left(1-B_{m,i,j}\right)\times M+TES_{m,i}-TES_{m,j}\geq t_{1},\forall m\in M,i,j\in I_{m},i\neq j.\\ I_{k,l}\times M+TSS_{2,l}-TES_{1,k}\geq t_{2},\forall k\in I_{1},\forall l\in I_{2}.\\ \left(1-I_{k,l}\right)\times M+TSS_{1,k}-TES_{2,l}\geq t_{2},\forall k\in I_{1},\forall l\in I_{2}.\\ B_{m,i,j},I_{k,l}\;\mathrm{is}\;a\;0,1\;\mathrm{variable} \end{array}$$

#### 3.2.2. Improved Scheduling Optimization Model Based on Variable Speed

**Figure 4:**
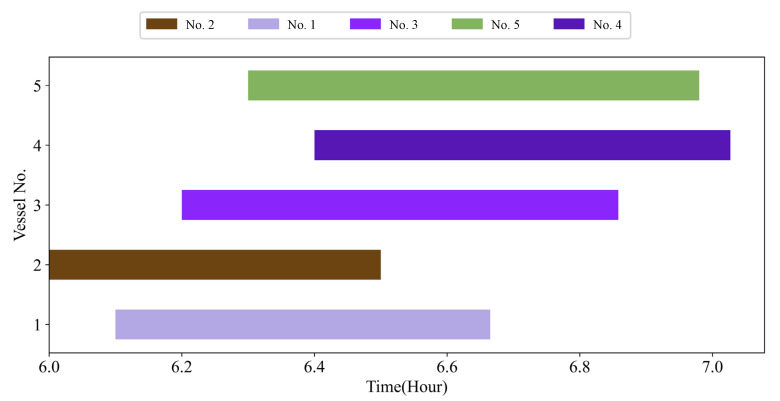
Optimal Gantt for the sample instance.

**Figure 5:**
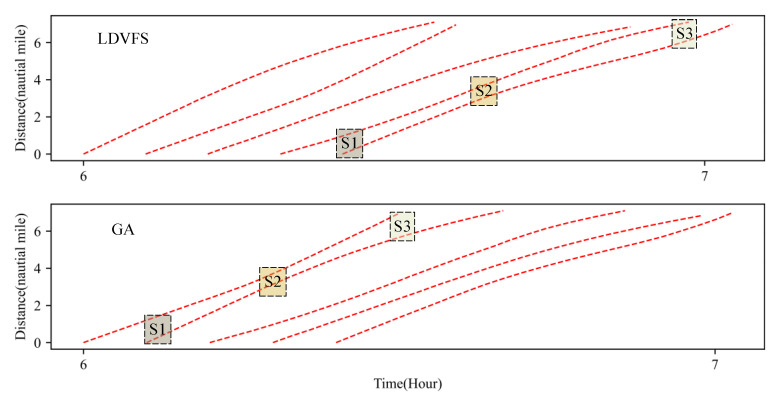
Optimal trajectories for the sample instance.

Based on the scheduling optimization model Formula (22), the optimal schedule result of the sample in Table 3 is shown in Table 6, which includes different results optimized by different priorities. Figure 4 shows the Gantt diagram of the best solution by GA. Figure 5 shows the diagram of temporal-spatial trajectories optimized by LDVFS and GA, where S1, S2, and S3 indicate the different stages of a voyage. From Figure 5, we can find that due to the existence of variable speed, if just considering the safety threshold at the entrance and exit points of the channel (stage S1 and stage S3) shown as Formula (22) instead of the whole process, the safety distance in stage S2 cannot be ensured.

To tackle this problem, considering variable speed, an improved scheduling optimization model was constructed. Because the trajectory of a ship continues, the safe navigation distance therefore needs to guarantee the whole process during sailing in the channel, not just in the entrance and exit shown as in Constraints (14–17) and (20–21).

**Figure 6:**
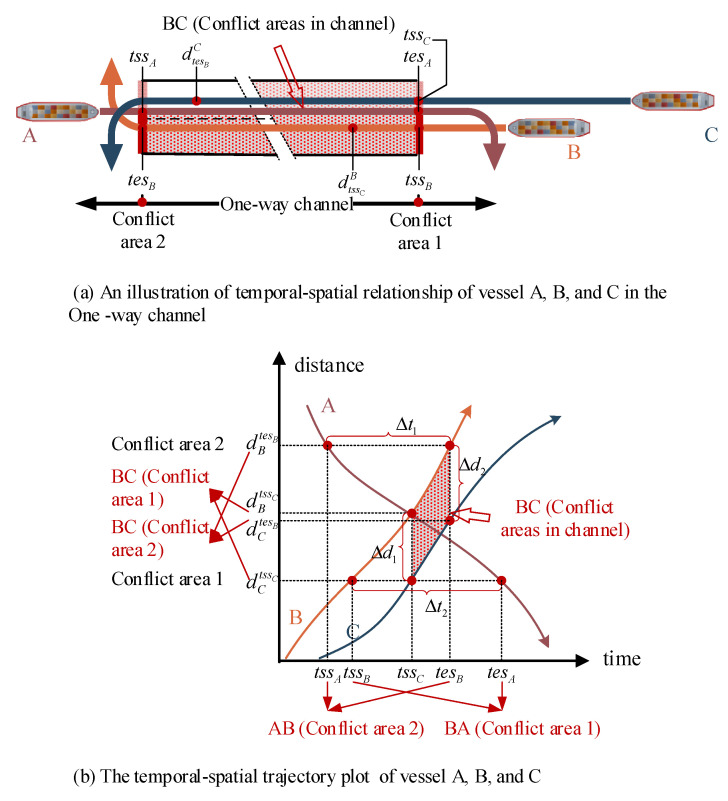
An illustration of vessels navigating with variable speed in a one-way channel.

Considering the actual trajectories of vessels, the safety requirement between any vessels can be illustrated by the temporal-spatial trajectory shown in Figure 6, which is an illustration of vessels navigating with variable speed in a one-way channel. Since the speed is variable when vessels are sailing in the channel, the vessel should therefore satisfy the minimum safety spacing requirements at any time in the same direction instead of just satisfying the safety requirement at the entrance and exit of the channel as in conflict areas 1 and 2 in Figure 6a, where vessel B and vessel C are sailing in the same direction, and vessel A is sailing in the opposite direction. When determining the sequence, as for those in the same direction as shown with vessel B and vessel C in Figure 6, the distance of the conflict areas in the channel between adjacent vessels needs to be greater than the safety threshold. As for those vessels in opposite direction as shown in vessel A and vessel B in Figure 6, the time difference $\Delta t_{1}=tes_{B}-tss_{A}$ and $\Delta t_{2}=tss_{B}-tes_{A}$ needs to have the same sign. The distance in all conflict areas between vessels at the same direction can be expressed as Equation (25), which is needed to be greater than the safety requirement set as 6 times the length of the vessel i under the same movement type, where $d_{m,i}^{t}$ denotes the sailing distance of the vessel $i,i\in I_{m}$ from the entrance of the channel, and ${\dot{d}}_{m,i}^{t}$ is the sailing speed of the vessel $i,i\in I_{m}$ at the time t in the planning horizon T. As for vessels in opposite directions, the safety threshold is set as 12 min (0.2 h), which can be constrained by Formula (24).

(23) $$\left\{\begin{array}{l} t_{-}=\max\left(tss_{m,i},tss_{m,i-1}\right)\\ t_{+}=\min\left(tes_{m,i},tes_{m,i-1}\right)\\ \int_{t-}^{t+}\left({\dot{d}}_{m,i-1}^{t}-{\dot{d}}_{m,i}^{t}\right)dt\geq 6\times L_{m,i},\forall m\in M,i\in I_{m} \end{array}\right.$$

(24) $$\left\{\begin{array}{l} tes_{1,i}-tss_{2,j}\geq 0.2\\ tss_{2,j}-tes_{1,i}\geq 0.2 \end{array}\right.,\forall i\in I_{1},j\in I_{2}$$

The speed of the vessel can be expressed at any time.

(25) $${\dot{d}}_{m,i}\left(t\right)=\frac{d_{m,i}\left(t\right)}{dt},\forall t\in \left[t_{m,i}^{-},t_{m,i}^{+}\right],m\in M,i\in I_{m}.$$

The initial and the termination state should satisfy the following constraints, where $v_{m,i}^{0}$ is the start sailing speed of the vessel $i,i\in I_{m}$,$v_{m,i}^{f}$ is the end sailing speed of the vessel $i,i\in I_{m}$, and L is the length of the channel.

(26) $$d_{m,i}\left(t_{m,i}^{-}\right)=0,{\dot{d}}_{m,i}\left(t_{m,i}^{-}\right)=v_{m,i}^{0},\forall m\in M,i\in I_{m}.$$

(27) $$d_{m,i}\left(t_{m,i}^{+}\right)=L,{\dot{d}}_{m,i}\left(t_{m,i}^{+}\right)=v_{m,i}^{f},\forall m\in M,i\in I_{m}.$$

We assume δ as a sufficiently small positive constant, then $t_{m,i}^{-}\leq k\delta \leq t_{m,i}^{+},k_{m,i}^{-}\delta =t_{m,i}^{-},k_{m,i}^{+}\delta =t_{m,i}^{+}$. The formula (26) can be discretized as follows.

(28) $${\dot{d}}_{m,i}\left[k\right]=\frac{d_{m,i}\left[k\right]-d_{m,i}\left[k-1\right]}{\delta },\forall k\in \left[k_{m,i}^{-}+1,k_{m,i}^{+}\right],m\in M,i\in I_{m}.$$

The safety distance of adjacent vessels in Constraint (23) can be transformed as follows.

(29) $$d_{m,i}\left[k\right]-d_{m,i-1}\left[k\right]\geq 6\times L_{m,i},\forall k\in \left[k_{m,i}^{-}+1,k_{m,i}^{+}\right],m\in M,i\in I_{m}.$$

**Figure 7:**
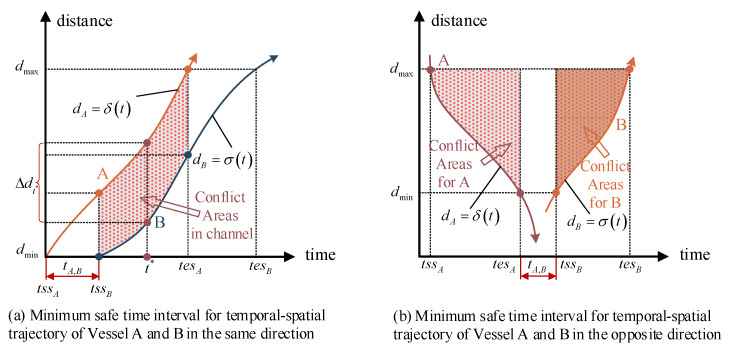
The minimum safe time window between two vessels.

Considering that formula (23) is non-linear, it is transformed into a discrete type defined as Formula (29). Based on Figure 6, the minimum safety time interval (MSTI) was defined as $t_{A,B}$ shown in Figure 7, which can ensure the adjacent vessels (A and B) have the safety distance in the entire conflict area both in the same direction and the opposite direction. The safety distance Constraint (29) is expressed as Formula (30) by introducing the parameter MSTI. In this paper, we just need to obtain the value of MSTI, where $t_{i,j}$ is the time threshold which can ensure that the distance of adjacent vessels satisfies the safety requirements.

Utilizing the same transform method as Constraint (9), Constraint (30) can be transformed as Formulas (31)–(32) by introducing 0−1 variables $I_{i,j}$.

(30) $$\left\{\begin{array}{cc} TSS_{i}-TSS_{j}\geq t_{j,j}, & if\;I_{i,j}=1\\ TSS_{j}-TSS_{i}\geq t_{i,j}, & if\;I_{i,j}=0 \end{array}\right.,\forall m\in M,i,j\in I=\underset{m\in M}{\cup }I_{m},i\neq j.$$

(31) $$I_{i,j}\times M+TSS_{j}-TSS_{i}\geq t_{i,j},\forall i,j\in I=\underset{m\in M}{\cup }I_{m},i\neq j.$$

(32) $$\left(1-I_{i,j}\right)\times M+TSS_{i}-TSS_{j}\geq t_{j,i},\forall i,j\in I=\underset{m\in M}{\cup }I_{m},i\neq j.$$

Above all, the model for vessel scheduling optimization based on speed variable can be shown as Formula (33), where the constraints are linear; therefore, this model consists of mixed-integer linear programming (MILP) that can be optimized by CPLEX solution solver of MATLAB or PYTHON.

(33) $$\begin{array}{l} \min\Gamma =\sum_{i\in I}\left(TSS_{i}-ETA_{i}\right)/\left|I\right|\\ s.t.TSS_{i}-TW_{i}^{l}\geq 0\\ TSS_{i}-ETA_{i}\geq 0\\ TSS_{i}-TW_{i}^{l}\geq 0\\ TW_{i}^{r}-TES_{i}\geq 0\\ I_{i,j}\times M+TSS_{j}-TSS_{i}\geq t_{i,j}\\ \left(1-I_{i,j}\right)\times M+TSS_{i}-TSS_{j}\geq t_{j,i}\\ I_{i,j}\;\mathrm{is}\;a\;0,1\;\mathrm{variable},\;\forall i,j\in I=\underset{m\in M}{\cup }I_{m},i\neq j \end{array}$$

## 4. Solution Approach

The optimization time of traditional commercial solvers (such as CPLEX) is directly proportional to the size of the problem; that is, as the problem size increases, the optimization time will also increase. Besides, the ship scheduling problem is a daily problem. In reality, it needs to be solved frequently considering the influence of the port operating environment, such as vessels requiring entry the port early, postponing port entry, and cancelling plans, etc. Therefore, a solution method that can provide an acceptable solution in reasonable computational times is required. To tackle this problem, on the one hand, three common-priority rule policies [35] were provided to verify the accuracy of the optimization results.

- First-in first-out (FIFO): a priority-based scheduling method and one of the manual scheduling methods. Specifically, the order of entry and exit is arranged according to the time of arrival at the port; that is, the ship that arrives first has the priority to use the channel.
- Large draft vessel first-served (LDVFS): a priority-based scheduling method and one of the manual scheduling methods. The sequence for in-wharf vessels and out-wharf vessels is arranged based on the draft. Generally, ships with larger drafts have poor maneuverability, and ports should pay more attention to them. Therefore, some ships with larger drafts are given priority to dispatch.
- Random served (RS): the sequence of both in-wharf vessels and out-wharf vessels is generated randomly. The rationale behind this is that the operator can dynamically determine the sequence of ships entering and leaving the channel without adopting a specific strategy.

On the other hand, we proposed an approach to solve the vessel scheduling problem considering variable speed (VSP*) which is based on our original GA-based vessel scheduling system (GA-VSS). Here, we briefly introduce the solution approach for VSP*.

Genetic algorithm is a popular heuristic algorithm in terms of the objective function value. It has been utilized to tackle many vessel scheduling optimization problems [9,10,15,17,18]. In this paper, single-layer real number initialization is utilized. Each individual is represented as a series of numbers, where the number on the chromosome is the assigned order. The initial population is formed by all those individuals, which are randomly generated. The advantage of this encoding and initialization method is that it is easy to encode the scheduling plan into the chromosome, and it is easy to decode and understand. The partially-matched crossover (PMX) method is used as the crossover operator. The roulette wheel selection criterion is used in GA-VSS. For a mutation operator, we combine three methods including local reverse mutation, exchange mutation, and insertion mutation. In each iteration, when a mutation operation is required, one of the above three methods is randomly selected. As for the illegal solution, we adopt the category by introducing a penalty function as formula (34), where a certain penalty is imposed on the illegal solution by adding a significant large positive constant M. Elite selection is utilized to select the top N individuals with the best fitness from the parent and offspring after the crossover operation and the mutation operation, where N is the number of both in-wharf vessels and out-wharf vessels.

(34) $$\Gamma =M+\sum_{i\in I}\left(TSS_{i}-ETA_{i}\right)/\left|I\right|,I=\underset{m\in M}{\cup }I_{m}$$

## 5. Numerical Simulation and Analysis

### 5.1. Simulation Setting

In this study, instances of different scales were randomly generated with the simulation data list in Table 7. As shown in Table 8, 13 instances were randomly generated from 18 vessels based on the index combination. We have conducted 13 sets of experiments with 5 ships, 10 ships, 15 ships, and 18 ships, respectively, to verify the effectiveness of the proposed model and the designed solution algorithm.

**Figure 8:**
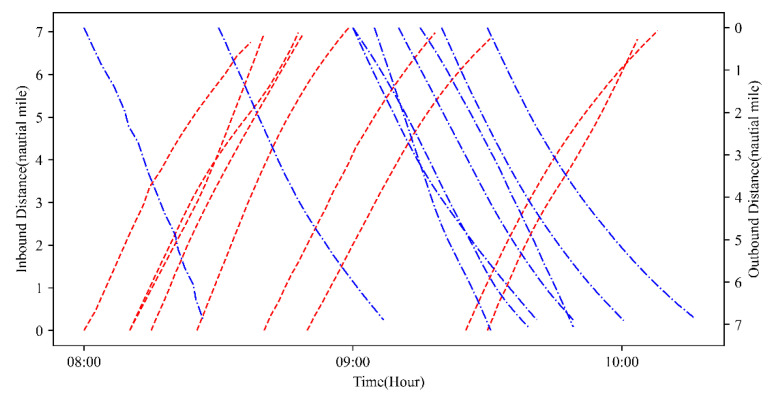
Temporal−spatial trajectories of 18 vessels.

In Table 7, the number of experimental vessels is 18. The basic data simulates the situation of the main channel of Tianjin Port, which is affected by the tide height. In this study, we assume the channel can only be accessed through one-way navigation. The tide data uses the hourly tide height data of a certain day in Tianjin Port, shown in Table 9. Figure 8 shows all trajectories of in-wharf vessels and all trajectories of out-wharf vessels during the channel, where all the sailing speeds of the vessels are different, and all of the space−time trajectories start at the estimated time of arrival.

The relevant parameter settings of GA are as follows: the number of individual vessels in the population is set to the number of vessels in different instances, the population size is set to 10, the mutation probability is 0.8, the maximum number of iterations is set to 2000, the maximum number of iterations is set to 300 when the optimal solution is continuous, and the maximum value M is set to 1000.

### 5.2. Data Preprocessing

**Figure 9:**
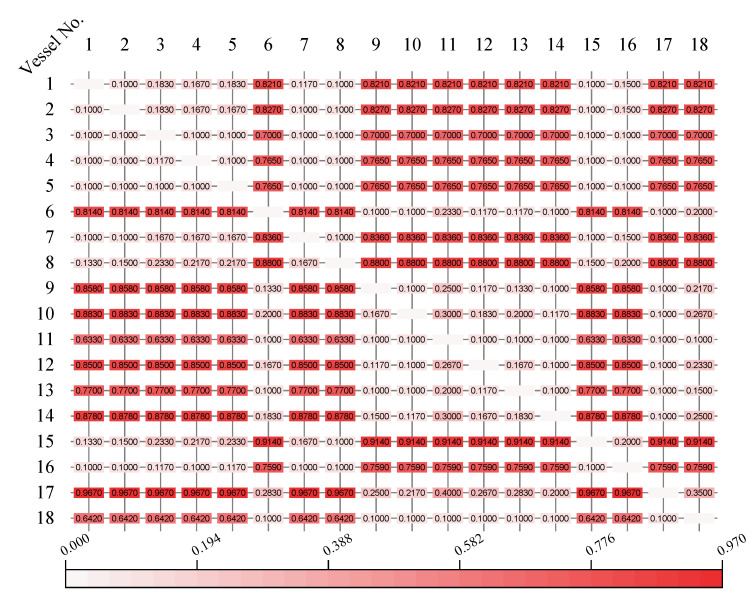
The minimum safe time interval of 18 vessels.

**Figure 10:**
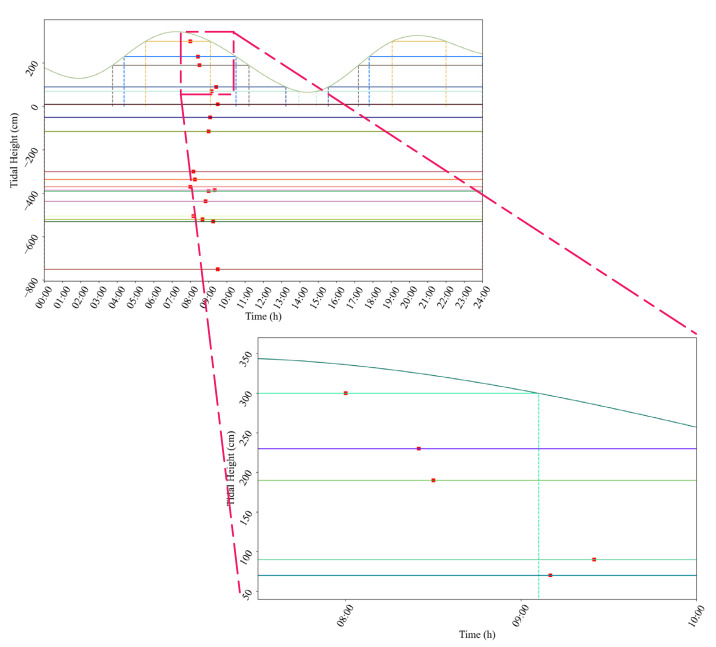
Ship navigable time window under the constraint of tide height.

With analysis of the proposed optimal scheduling model, we find that we need to preprocess the above basic data. According to our original GA-based vessel scheduling system (GA-VSS), the result of data processing is shown as follows. Figure 9 is the value of MSTI between any two vessels in Table 7, where the detailed data are listed in Appendix A Table A1. The pseudocode for calculating the value of MSTI is presented in Appendix A Table A2. Figure 10 shows the navigable time windows under the constraint of tide height, where the detailed data is listed in Table 10. The line segment in Figure 10 represents the navigable time windows of the vessels, and the red point indicates the estimated time of arrival.

### 5.3. Comparison between Different Methodologies

As mentioned before, the improved vessel scheduling optimization model is MILP; therefore, it can be directly solved by commercial solution solver CPLEX. In the following, we would analyze the effectiveness of the VSP model considering the variable speed with different optimal scheduling methods.

The final solution obtained by the GA method is shown in Table 11. Comparing it to the solution in Table 12, we can observe that the proposed GA method can also obtain a result similar to the final solution obtained by the CPLEX solver, where the relative error was smaller than other methods.

**Figure 11:**
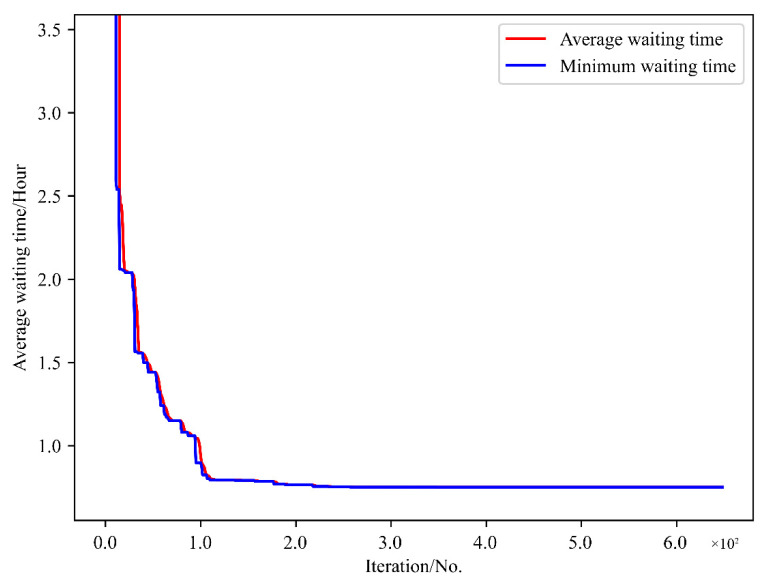
Iteration convergence diagram of instance “Inst_18_1”.

**Figure 12:**
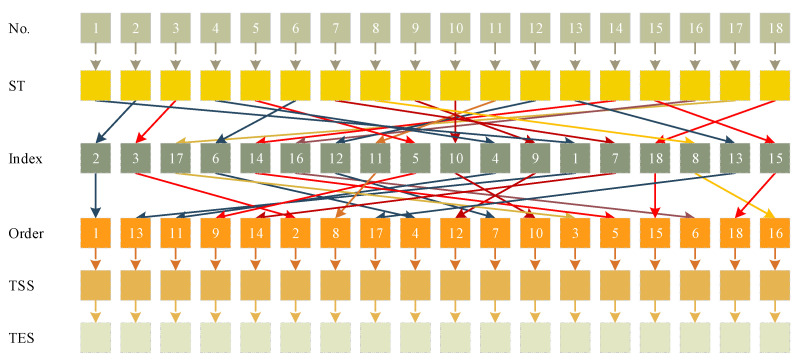
Parallel coordinate graph of experimental result.

**Figure 13:**
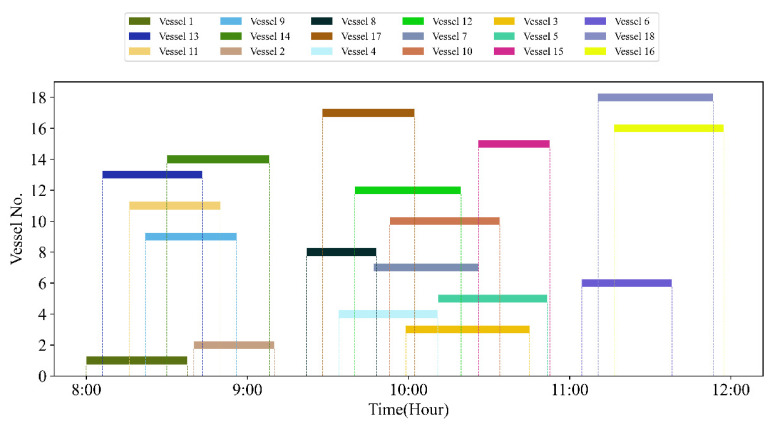
Gantt diagram of the optimization result of “Inst_18_1”.

**Figure 14:**
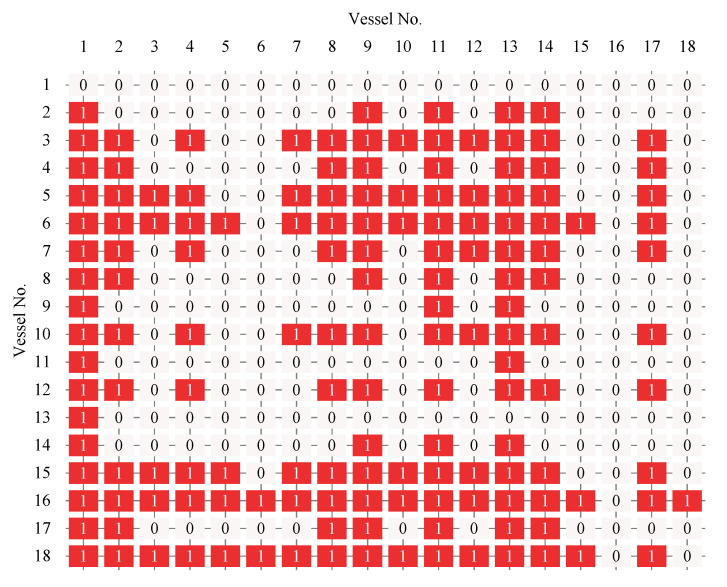
The result of decision variable of instance “Inst_18_1”.

**Figure 15:**
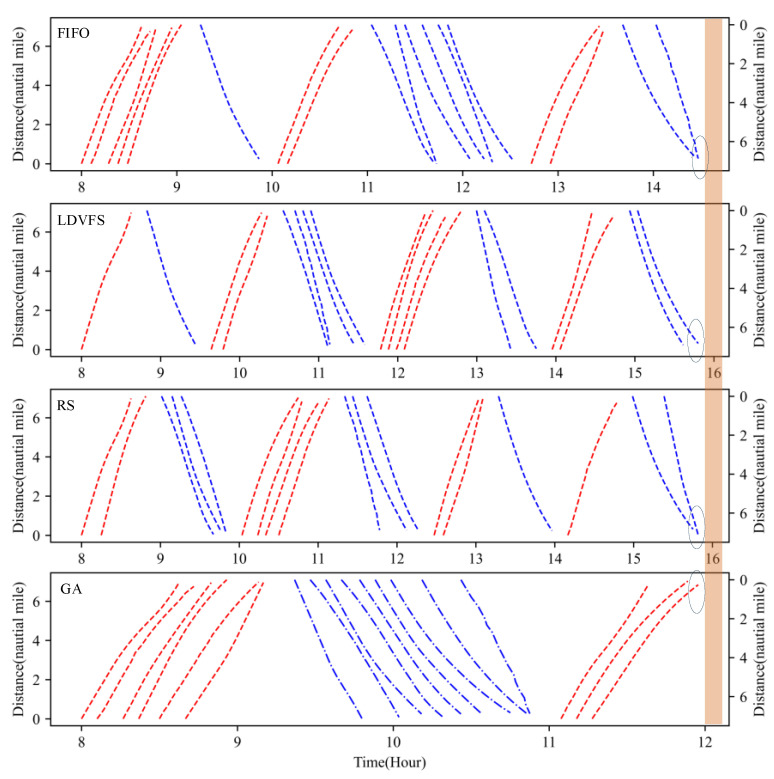
Space−time trajectories of the solutions of different heuristics.

Figure 11 is the iteration convergence diagram of instance “Inst_18_1” including the minimum waiting time and the average time for waiting of each generation, where the iteration ends at generation 648, and the value of the best solution remains unchanged at generation 348. To better understand the relationship between the optimization results of Table 11, the parallel coordinate graph of the optimal result was presented in Figure 12, which indicates the connection between each attribute, such as sailing time, the assigned order, the selected index of vessels, the vessels’ numbers, etc. For example, the third attribute refers to the index of the ship, and its value corresponds to ship number one to one. The fourth attribute is the numerical value corresponding to the optimization solution after the third attribute is renumbered from left to right to 1–18. Figure 13 is the Gantt diagram of the optimization result of instance “Inst_18_1”, from which the schedule of vessels can be identified. Figure 14 is the result of the decision variable in the vessel scheduling optimization model. According to the 0–1 variables in each row or column of Figure 14, the final ship dispatch sequence can be obtained. Figure 15 shows the space−time trajectories of the optimal solution of instance “Inst_18-1”, where the conversion status and the makespan can also be obtained. Compared with Figure 5, it can in the future validate the advantage of the indeterminate speed model.

With regard to the stability of the proposed method, the comparison between different scales of instance was carried out as shown in Table 13. The proposed model was optimized by CPLEX solver, FIFO, LDVFS, RS, and GA method. The difference rate was expressed as ‘GAP’ (GAP = (near-optimal solution—exact solution)/ exact solution * 100%). Table 13 indicates that the solution obtained by the GA method has a higher quality for any scale of instances than the general scheduling method. Comparing the calculation time of the CPU in Table 12 and Table 13, it can be found that for small-scale problems, the proposed GA method is less efficient than CPLEX. However, for relatively large-scale problems, the proposed method is more efficient than CPLEX. Considering that hundreds of vessel dispatch operations should be carried out, the presented GA method will be more practical than CPLEX solver and other priority rules, and it also can reduce the waiting time of in-wharf vessels and out-wharf vessels.

## 6. Conclusions and Future Work

This research aims to minimize the average waiting time of the continuous vessel scheduling problem by considering the uncertain speed of vessels. Based on this, the proposed VSP model was simplified by introducing the concept of MSTI, which integrated the multiple time constraints of the model and reduced the complexity of understanding. The problem is first modeled as a MILP model and solved by GA within reasonable execution time. Numerical experimental comparative analysis is conducted to compare both the innovation between the indeterminate velocity model and fixed velocity model and the accuracy between CPLEX solver and GA. The contributions made in this study can be summarized in the following results—it can be concluded that:

- The designed heuristic solution method has higher optimization accuracy than general priority scheduling methods such as FIFO, LDVFS, RS, etc. Besides, it can obtain the near-optimal solution for both small-scale instances and large-scale instances.
- Based on the proposed VSP model, the concept of MSTI was proposed for better understanding which reduced the number of constraints by integrating the time constraints of the proposed VSP model.
- The model built based on variable speed has obvious efficiency in optimization solutions. Compared with the fixed-speed scheduling model, it also has obvious practicality in spatio-temporal trajectory and can ensure the safety of the whole voyage.

To sum up, we revealed the safety constraint mechanism under the whole voyage in the continuous vessel scheduling model, simplified and linearized the proposed vessel model, and proposed a heuristic algorithm for improving the efficiency of optimal scheduling. This study not only can validate the advantages of the indeterminate speed model but also improve the working efficiency and provide decisions for port operators. In the future, such a method can continue to be applied to other targeted ports and waterways.

## Figures and Tables

**Table 1:** Contributions to related studies on the VSP.

No.	Citation	Waterway Structure				Safety Limit		Sailing Speed		Methodology
		One	Two	Multiple	Compound	TTW	Separation Dist/Time	Fixed	Variable	
1	Liu B. et al. (2021)	✓				✓	✓	✓		MIP + ALNS
2	Abou Kasm et al. (2021)	✓					✓	✓		MIP + CS
3	Lübbecke, E. (2019)		✓				✓	✓		LS + RH
4	Muñuzuri, J. (2018)	✓				✓	✓	✓		ES
5	Günther, E. (2010, 2011)		✓				✓	✓		MIP
6	Meisel, F. (2019)		✓				✓	✓		MIP
7	Andersen, T. (2021)		✓				✓	✓		MIP + ICAM
8	Guo, Z. (2021)	✓				✓	✓	✓		MIP
9	Lalla-Ruiz et al. (2018)			✓		✓	✓	✓		MIP; MIP + SA
10	Hill et al. (2019)			✓		✓	✓	✓		MIP + SA
11	Zhang B. et al. (2019, 2020, 2021)	✓	✓		✓	✓	✓	✓		GA
12	Li J. et al. (2020)			✓		✓	✓	✓		TS + NSGA-II
13	Elena Kelareva (2012)	✓				✓	✓	✓		CP + MIP
14	Li and Jia (2019)		✓			✓		✓		MIP + CG
15	Zhang X. et al. (2016, 2017, 2019)	✓			✓		✓	✓		SA + GA/MOGA
16	Our work	✓				✓	✓		✓	MILP + GA

Notes: “TTW”: tidal time window; “MIP”: mixed integer programming; “ALNS”: adaptive large neighborhood search; “CS”: constraint separation; “LS”: local search; “RH”: rolling horizon heuristic; “ES”: exhaustive search; “ICAM”: iterative conflict-adding matheuristic; “SA”: simulated annealing; “GA”: genetic algorithm; “TS”: tabu search; “NSGA-II”: nondominated sorting genetic algorithms; “CP”: constraint programming; “CG”: column generation; “MOGA”: multi-objective genetic algorithm.

**Table 2:** Sample vessel plan.

Ship Name	MMSI	IMO	Call sign	Flag	Ship Type	Length	Width	Draft
YANTIAN	256930000	9305594	9HA4039	Malta	Bulk	350	43	6.4
MSC RIFAYA	636017685	9767388	D5MF9	Libya	Container	85	12.8	6.2
COSCO UNIVERSE	477157400	9795610	VRRP4	Hong Kong	Container	400	59.0	12.8
**State**	**Start position**	**Stop position**	**ETA**	**Start time**	**End time**	**Channel**	**Online point**	**Offline point**
Out	S5	O	2018/3/13 13:00	2018/3/13 13:00	2018/3/13 13:00	One-way	Beacon 1	5
In	A1	N1	2018/3/13 13:00	2018/3/13 13:00	2018/3/13 13:00		Beacon1	3
Move	D34	D4	2018/3/13 13:30	2018/3/13 13:30	2018/3/13 13:30		Beacon1	4

**Table 3:** Sample general VSP instance.

Vessel No.	Length (m)	Draft (m)	Fixed Speed (m/s)	ETA	Tidal Time Window	State
1	365.5	14.44	6.37	6:00	[3:48,11:08]	In
2	265.98	12.36	7.20	6:00	[0:00,24:00]	In
3	125.0	6.13	5.47	6:00	[0:00,24:00]	Out
4	115.02	6.00	5.74	6:00	[0:00,24:00]	Out
5	170.0	8.83	5.30	6:00	[0:00,24:00]	Out

**Table 4:** VSP optimization sequence analysis.

Index	Schedule Solution
1	1→2→3→4→5
2	1→2→3→5→4
3	1→2→4→3→5
4	1→2→4→5→3
5	1→2→5→3→4
6	1→2→5→4→3

**Table 5:** Notation.

Sets and Parameters
M: Set of movement types: M={m:m=1,IN;m=2,OUT} $I_{m}$: Set of in-wharf vessels or out-wharf vessels under movement m. Indices $i,j\in I_{m}=\left\{1:\left|I_{m}\right|\right\}$, where $\left|I_{m}\right|$ denotes the number of vessels under movement m T: Set of periods in the planning horizon, indexed by t $ETA_{m,i}$: The estimated time of arrival of the vessel $i,i\in I_{m}$ under movement m $TES_{m,i}$: The time of end schedule for the vessel $i,i\in I_{m}$ under movement m $\Delta_{m,i}$: Sailing time for the vessel $i,i\in I_{m}$ under movement m $V_{m,i}$: Sailing speed for the vessel $i,i\in I_{m}$ under movement m $L_{m,i}$: Length of the vessel $i,i\in I_{m}$ under movement m $TW_{m,i}^{l}$: The lower bound of the navigable time window of the ship $i,i\in I_{m}$ under movement m constrained by draft and tidal height $TW_{m,i}^{r}$: The upper bound of the navigable time window of the ship $i,i\in I_{m}$ under movement m constrained by draft and tidal height $t_{1}$: Safe navigation interval between vessels in the same direction $t_{2}$: Safe navigation interval between vessels in the opposite direction $t_{i,j}$: Safe navigation interval between vessels $i,j\in I=\underset{m\in M}{\cup }I_{m},i\neq j$ M: A sufficiently large positive constant **Decision Variables** D: Total waiting time for all in-wharf vessels and out-wharf vessels from the vessel arrival or departure time Γ: Average waiting time for all in-wharf vessels and out-wharf vessels $TSS_{m,i}$: The time of start scheduling for the vessel $i,i\in I_{m}$ under movement m $B_{m,i,j}$: 0–1 variable, 0 if vessel $i,i\in I_{m}$ precedes $j,j\in I_{m}$ in the sequence under movement m; 1 otherwise $I_{k,l}$: 0–1 variable, 0 if vessel $k,k\in I_{1}$ precedes $l,l\in I_{2}$ in the sequence; 1 otherwise

**Table 6:** Sample VSP instance and its optimal solution.

Mode	Vessel Scheduling Sequence	Waiting Time (h)
FIFO	1→2→3→4→5	0.2
LDVFS	1→2→5→3→4	0.21
RS	4→3→1→5→2	0.31
CPLEX	2→1→3→5→4	0.2
GA	2→1→3→5→4	0.2

**Table 7:** Basic data of ship scheduling optimization.

No.	Length (m)	State	ETA	Tidal Time Windows	Draft (m)	UKC (m)
1	184.95	In	8:00:00	(0.0, 24.0)	7.50	1.30
2	292.00	In	8:00:00	((5.55, 9.1), (19.05, 22.0))	13.90	1.60
3	228.99	In	8:10:00	(0.0, 24.0)	6.39	1.06
4	304.60	In	8:10:00	(0.0, 24.0)	8.60	0.90
5	183.00	In	8:15:00	(0.0, 24.0)	8.20	0.94
6	292.00	Out	8:25:00	((4.37, 10.5), (17.79, 24.0))	13.30	1.50
7	229.00	In	8:30:00	((3.74, 11.21), (17.2, 24.0))	12.90	1.50
8	159.99	In	8:40:00	(0.0, 24.0)	6.50	0.80
9	249.90	Out	8:50:00	(0.0, 24.0)	7.20	0.93
10	294.00	Out	9:00:00	(0.0, 24.0)	10.25	1.10
11	182.50	Out	9:00:00	(0.0, 24.0)	7.60	1.00
12	330.00	Out	9:05:00	(0.0, 24.0)	10.80	1.20
13	291.98	Out	9:10:00	((0.0, 13.94), (14.9, 24.0)	11.90	1.30
14	189.99	Out	9:15:00	(0.0, 24.0)	6.40	0.80
15	294.12	In	9:20:00	(0.0, 24.0)	7.80	0.85
16	330.00	In	9:25:00	((0.0, 13.23), (15.55, 24.0))	12.00	1.40
17	116.85	Out	9:30:00	(0.0, 24.0)	4.50	0.50
18	225.00	Out	9:30:00	(0.0, 24.0)	11.20	1.40

**Table 8:** Experimental case.

Case	Vessel Index Combination
Inst_5_1	17,5,3,13,8
Inst_5_2	18,11,9,8,16
Inst_5_3	3,13,10,16,6
Inst_5_4	17,18,1,9,8
Inst_10_1	11,8,13,9,5,1,17,18,14,3
Inst_10_2	2,10,12,7,13,11,15,3,9,16
Inst_10_3	6,10,14 18 13,2,11,9,4,5
Inst_10_4	6,8,4,10,9,2,16,14,3,17
Inst_15_1	6,5,18,1,4,12,3,15,13,7,10,2,14,11,9
Inst_15_2	18,6,12,10,2,4,14,9,8,7,13,1,15,17,5
Inst_15_3	15,17,2,4,6,8,12,16,5,1,7,11,18,13,10
Inst_15_4	2,14,15,8,1,7,12,13,16,4,3,18,17,6,11
Inst_18_1	2,3,17,6,14,16,12,11,5,10,4,9,1,7,18,8,13,15

**Table 9:** Hourly tide height data for a certain day in Tianjin Port.

**Time**	**0000**	**0100**	**0200**	**0300**	**0400**	**0500**	**0600**	**0700**	**0800**	**0900**	**1000**	**1100**
TH/cm	179	143	130	153	206	270	320	344	336	304	257	202
**Time**	**1200**	**1300**	**1400**	**1500**	**1600**	**1700**	**1800**	**1900**	**2000**	**2100**	**2200**	**2300**
TH/cm	146	99	69	72	112	176	244	298	324	323	300	268

**Table 10:** Navigable time windows constrained by tidal height.

No.	NTW (H)
2	((5.55, 9.1), (19.05, 22.0))
6	((4.37, 10.5), (17.79, 24.0))
7	((3.74, 11.21), (17.2, 24.0))
13	((0.0, 13.94), (14.9, 24.0))
16	((0.0, 13.23), (15.55, 24.0))

**Table 11:** Optimal results of 18 vessel experiment using GA.

No.	ST	Index	Order	TSS	TES
1	0.627	2	1	8.0	8.627
2	0.500	3	13	8.1	8.721
3	0.767	17	11	8.267	8.832
4	0.614	6	9	8.367	8.932
5	0.678	14	14	8.5	9.136
6	0.559	16	2	8.667	9.167
7	0.65	12	8	9.367	9.8
8	0.433	11	17	9.467	10.037
9	0.565	5	4	9.567	10.181
10	0.683	10	12	9.667	10.325
11	0.565	4	7	9.784	10.434
12	0.658	9	10	9.884	10.567
13	0.621	1	3	9.984	10.751
14	0.636	7	5	10.184	10.862
15	0.442	18	15	10.434	10.876
16	0.680	8	6	11.076	11.635
17	0.570	13	18	11.176	11.89
18	0.714	15	16	11.276	11.956

**Table 12:** Comparisons between CPLEX, FIFO, LDVFS, RS, and GA of instance “Inst_18_1”.

Mode.	Vessel Scheduling Sequence	CPU (s)	Waiting Time (h)	GAP (%)
Select	2,3,17,6,14,16,12,11,5,10,4,9,1,7,18,8,13,15	--	--	--
CPLEX	1,13,11,9,2,14,16,8,15,17,4,12,7,10,5,3,6,18	77.531	0.702	<0.0089%
FIFO	1,13,2,11,9,4,14,16,12,8,10,7,17,5,18,6,3,15	--	--	--
LDVFS	1,4,14,6,17,15,7,10,11,9,13,18,8,12,2,16,5,3	--	2.930	317.3789%
RS	1,9,12,4,17,18,6,16,14,15,10,7,11,2,5,13,3,8	--	2.434	246.7236%
GA	1,13,11,9,14,2,8,17,4,12,7,10,3,5,15,6,18,16	7.074	0.750	6.83760%

**Table 13:** Comparisons between CPLEX, FIFO, LDVFS, RS, and GA.

Case	CPLEX	CPU (s)	GAP	GA	CPU (s)	GAP	FIFO	GAP	LDVFS	GAP	RS	GAP
Inst_5_1	0.11	0.016	0%	0.11	1.12	0%	0.11	0%	1.31	1091%	1.12	918%
Inst_5_2	0.48	0.016	0%	0.48	0.98	0%	0.85	78%	1.22	156%	1.83	285%
Inst_5_3	0.21	0.000	0%	0.21	1.06	0%	0.21	0%	1.06	413%	0.73	252%
Inst_5_4	0.22	0.015	0%	0.22	1.06	0%	0.27	22%	2.13	849%	1.21	439%
Inst_10_1	0.28	0.031	0%	0.29	2.33	3%	0.46	63%	2.32	719%	2.24	690%
Inst_10_2	0.45	0.031	0%	0.45	2.65	0%	0.64	42%	1.72	282%	1.88	317%
Inst_10_3	0.25	0.047	0%	0.26	2.38	4%	0.32	31%	1.29	425%	0.51	107%
Inst_10_4	0.41	0.032	0%	0.41	2.46	0%	1.25	202%	2.66	543%	1.45	250%
Inst_15_1	0.54	20.422	0%	0.55	6.14	1%	1.58	192%	2.69	397%	2.31	328%
Inst_15_2	0.59	1.703	0%	0.59	7.68	0%	1.68	187%	2.74	367%	2.61	345%
Inst_15_3	0.62	0.781	0%	0.62	7.51	0%	1.70	174%	2.41	289%	1.69	173%
Inst_15_4	0.61	0.828	0%	0.70	11.01	15%	1.63	169%	2.66	338%	1.83	201%
Average	0.40	1.994	0%	0.41	3.865	2%	0.89	97%	2.01	489%	1.62	359%

## Data Availability

Not applicable.

## Appendix A

**Table A1:** Pseudocode of calculating the value of MSTI.

Pseudocode of Calculating the Value of MSTI
Input the basic data of vessels $P=\left[MMSI,IO,LEN,ST,NTW\right]$ which includes the identified number *MMSI*, movement type *IO*, length *LEN*, sailing time *ST*, and the navigable time windows *NTW*; the threshold of safety distance $d_{s}$; the index of the case $C_{s}$; the optimal scheduling sequence $O_{s}$;$tr_{i}=\left[\left(s_{0},t_{0}\right),\left(s_{1},t_{1}\right),\cdots ,\left(s_{n},t_{n}\right)\right]$; the track of vessels $Tr=\left[t_{1},t_{2},\cdots ,t_{n}\right]$. $P_{s}\leftarrow \emptyset ,Tr_{s}\leftarrow \emptyset ,MSTI\leftarrow \emptyset$ For each $i\in C_{s}$ $[id_{s},io_{s},len_{s},st_{s},ntw_{s}]\leftarrow$ the data of P at the index *i* of $C_{s}$ $tr_{i}\leftarrow$ the data of Tr at the index *i* of $C_{s}$ $P_{s}$ append $\left[id_{s},io_{s},len_{s},st_{s},ntw_{s}\right]$ $Tr_{s}$ append $tr_{i}$ End for For each $j\in P_{s}$ $m\leftarrow O_{s}^{j}$ The value of $O_{s}$ at the index *j* $Tr_{s}^{m}\leftarrow$ the data $Tr_{s}$ at the index *m* $IO_{m}\leftarrow$ the movement type of vessels *m* msti←∅ For each $k\in P_{s}$ $n\leftarrow O_{s}^{k}$ The value of $O_{s}$ at the index *k* $Tr_{s}^{n}\leftarrow$ the data of $Tr_{s}$ at the index *n* $IO_{n}\leftarrow$ the movement type of vessels *n* If $j\neq k\&IO_{m}==IO_{n}$ then $t\leftarrow Func\left(Tr_{s}^{m},Tr_{s}^{n},d_{s}\right)$ Else t←0.2 msti append *t* End for MSTI append msti Return MSTI
$Func\left(Tr_{s}^{m},Tr_{s}^{n},d_{s}\right)$ $f_{1}\leftarrow$Fitting the track *m* $f_{2}\leftarrow$Fitting the track *n* msti←∅,T←60 For each t∈T $t_{1}^{-}\leftarrow$The start time of track m $t_{2}^{-}\leftarrow$The start time of track *n* $t_{1}^{+}\leftarrow$The end time of track m $t_{2}^{+}\leftarrow$The end time of track *n* $t_{\min}\leftarrow$The minimum time of $t_{1}^{+},t_{2}^{+}$ $d_{1}\leftarrow f_{1}\left(t_{1}^{-}+t\right)-f_{2}\left(t_{2}^{-}\right)$The step size of sailing time $d_{2}\leftarrow f_{1}\left(t_{\min}\right)-f_{2}\left(t_{\min}+t\right)$The step size of sailing time $s\leftarrow \left(t_{\min}-\left(t_{2}^{-}+t\right)\right)$The step size of sailing time If $s>1\cup d_{1}\geq d_{s}\cup d_{2}\geq d_{s}$ then msti←t End for Return msti

**Table A2:** MSTI.

No.	1	2	3	4	5	6	7	8	9	10	11	12	13	14	15	16	17	18
1	0.000	0.100	0.183	0.167	0.183	0.821	0.117	0.100	0.821	0.821	0.821	0.821	0.821	0.821	0.100	0.150	0.821	0.821
2	0.100	0.000	0.183	0.167	0.167	0.827	0.100	0.100	0.827	0.827	0.827	0.827	0.827	0.827	0.100	0.150	0.827	0.827
3	0.100	0.100	0.000	0.100	0.100	0.700	0.100	0.100	0.700	0.700	0.700	0.700	0.700	0.700	0.100	0.100	0.700	0.700
4	0.100	0.100	0.117	0.000	0.100	0.765	0.100	0.100	0.765	0.765	0.765	0.765	0.765	0.765	0.100	0.100	0.765	0.765
5	0.100	0.100	0.100	0.100	0.000	0.765	0.100	0.100	0.765	0.765	0.765	0.765	0.765	0.765	0.100	0.100	0.765	0.765
6	0.814	0.814	0.814	0.814	0.814	0.000	0.814	0.814	0.100	0.100	0.233	0.117	0.117	0.100	0.814	0.814	0.100	0.200
7	0.100	0.100	0.167	0.167	0.167	0.836	0.000	0.100	0.836	0.836	0.836	0.836	0.836	0.836	0.100	0.150	0.836	0.836
8	0.133	0.150	0.233	0.217	0.217	0.880	0.167	0.000	0.880	0.880	0.880	0.880	0.880	0.880	0.150	0.200	0.880	0.880
9	0.858	0.858	0.858	0.858	0.858	0.133	0.858	0.858	0.000	0.100	0.250	0.117	0.133	0.100	0.858	0.858	0.100	0.217
10	0.883	0.883	0.883	0.883	0.883	0.200	0.883	0.883	0.167	0.000	0.300	0.183	0.200	0.117	0.883	0.883	0.100	0.267
11	0.633	0.633	0.633	0.633	0.633	0.100	0.633	0.633	0.100	0.100	0.000	0.100	0.100	0.100	0.633	0.633	0.100	0.100
12	0.850	0.850	0.850	0.850	0.850	0.167	0.850	0.850	0.117	0.100	0.267	0.000	0.167	0.100	0.850	0.850	0.100	0.233
13	0.770	0.770	0.770	0.770	0.770	0.100	0.770	0.770	0.100	0.100	0.200	0.117	0.000	0.100	0.770	0.770	0.100	0.150
14	0.878	0.878	0.878	0.878	0.878	0.183	0.878	0.878	0.150	0.117	0.300	0.167	0.183	0.000	0.878	0.878	0.100	0.250
15	0.133	0.150	0.233	0.217	0.233	0.914	0.167	0.100	0.914	0.914	0.914	0.914	0.914	0.914	0.000	0.200	0.914	0.914
16	0.100	0.100	0.117	0.100	0.117	0.759	0.100	0.100	0.759	0.759	0.759	0.759	0.759	0.759	0.100	0.000	0.759	0.759
17	0.967	0.967	0.967	0.967	0.967	0.283	0.967	0.967	0.250	0.217	0.400	0.267	0.283	0.200	0.967	0.967	0.000	0.350
18	0.642	0.642	0.642	0.642	0.642	0.100	0.642	0.642	0.100	0.100	0.100	0.100	0.100	0.100	0.642	0.642	0.100	0.000
